# Pyogenic Liver Abscess Presenting as an Initial Manifestation of Underlying Hepatocellular Cancer: A Case Report in Ethiopia

**DOI:** 10.4314/ejhs.v32i3.24

**Published:** 2022-05

**Authors:** Hailemichael Desalegn, Ayantu Tesfaye, Paulos Shume

**Affiliations:** 1 St. Paul's Hospital Millennium Medical College, Addis Ababa, Ethiopia; 2 Menelik II Hospital, Addis Ababa, Ethiopia

**Keywords:** Liver Abscess, Hepatocellular Cancer, Ethiopia

## Abstract

**Background:**

An adult patient presented with right abdominal pain and fever to a primary care physician and abdominal ultrasound was performed. With an initial diagnosis of a liver abscess, he was discharged from the hospital after treatment with antibiotics and drainage of the collection. However, the patient had persistent clinical findings on the same site which was later confirmed as Hepatocellular cancer.

**Case Presentation:**

A 40 years old male patient who was known to have Type 2 Diabetes and Hypertension for 10 years on oral medications referred to the Gastroenterology/Hepatology unit with right upper quadrant pain, loss of appetite, nausea, vomiting of ingested matter, and significant weight loss. On further inquiry, he had been admitted six months back for similar complaints and was managed with antibiotics and drainage of an abscess collection.

The multi-phasic abdominal CT scan and raised alphafetoprotein confirmed Hepatocellular Cancer which initially has presented as a pyogenic liver abscess.

**Conclusion:**

Hepatocellular cancer should be suspected and early diagnosis should be made in individuals presenting with a liver abscess and having risk factors for liver cancer.

## Introduction

A 40 years old male patient presented with a rare presentation of Hepatocellular cancer with an initial manifestation of a liver abscess. He was appropriately treated for the liver abscess with drainage and provision of antibiotics. Due to lack of anticipation that he could have a liver cancer, proper follow-up was not implemented, and when he was evaluated after 6 months, he had a liver cancer on the same site where the abscess has been drained. On further work-up of potential risk factors, he was diagnosed to have Hepatitis C virus in addition to the type 2 DM which could predispose the development of a hepatocellular cancer. It is important that clinicians dealing with such patients should send them to an MDT (Multi-disciplinary team) for further work-up of patients with liver abscess.

## Case Presentation

A 40 years old male patient was known to have Type 2 DM for 10 years on oral medication which was changed to insulin for 2 weeks. He is also known to have Hypertension of the same duration on oral medications referred to the Gastroenterology/Hepatology unit with right upper quadrant pain, loss of appetite, nausea, vomiting of ingested matter and significant weight loss. The total duration of symptoms has been around 6 months, but he has worsening for 2 weeks.

**Figure 1:**
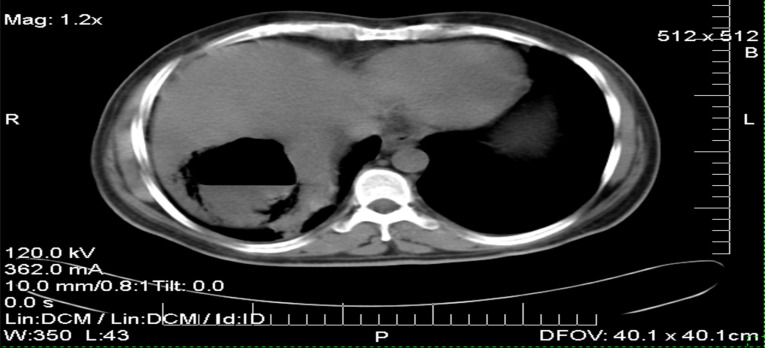
Tri-phasic abdominal CT scan on the initial presentation

On further inquiry, he had been admitted six months back for similar complaints and was managed with antibiotics and drainage of an abscess collection. During his previous admission, his liver chemistry showed an elevated AST of 160 mg/dl (elevated 4X), Alkaline phosphatase and other liver panels were unremarkable; an abdominal ultrasound suggested an echo containing mass 5X6 cm in the right lobe of the liver with an index of liver abscess; the CT imaging has revealed gasforming hepatic abscess (see Figure 1) for which ultrasound guided abscess drainage was done and around 70 cc of thick, yellowish pus was aspirated. Using seldinger technique, the drain was left in situ. He was then followed in the ward and after proper irrigation and antibiotics, he has shown improvement. He was treated with ceftriaxone, metronidazole and before discharge there were radiological improvement and sent home.

On current admission, his general appearance showed a chronically ill-appearing man with signs of undernutrition, prominent zygomatic bones and prominent ribs. The physical examination revealed tenderness on the right upper abdominal quadrant with palpable liver 2 cm below the right costal margin (total liver span of 14 cm); no splenomegaly or signs of fluid collection.

On laboratory investigation, Complete Blood Count (CBC) was within normal limits, ESR was 43 mm/hr on the referral and at repeat test it was 107 mm/hr, Renal function tests were normal, the coagulation profiles were within normal limits and on liver function tests, ALP was raised to 185 IU/l, Bilirubin (T) 2.28 mg/dl, ALT=75.7 (20–40), AST=89.8 (20–40), Albumin=2.70 g/dl (3.5–5.5), RBS=153 mg/dl, HIV serology was negative.

**Figure 2:**
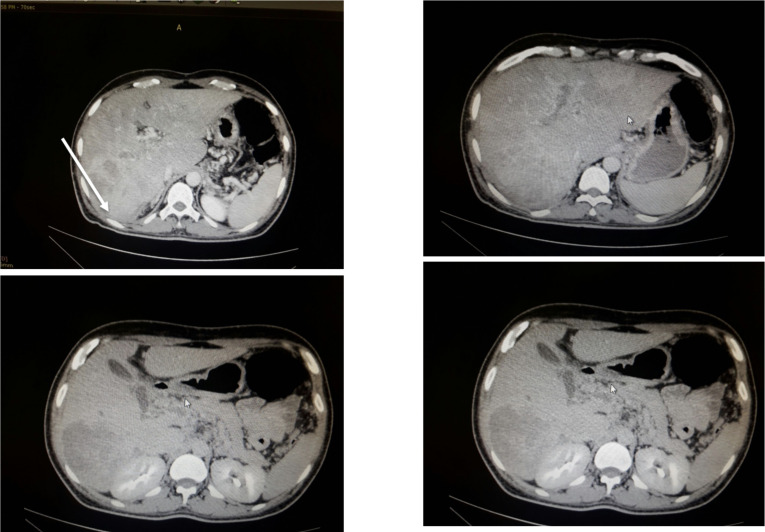
Tri-phasic abdominal CT scan 6 months after the initial abscess treatment (on the second admission to the Hospital)

A Tri-phasic abdominal CT scan revealed a hyper-vascular lesion with portal vein thrombosis and collaterals secondary to Hepatocellular carcinoma (See Figure 2). The size of the liver mass measures 91 mm X 94 mm with an adjacent satellite nodule located in segment VI and VII which shows avid enhancement and rapid washout in the venous phase. The portal vein has filling defect with peri-portal collaterals.

On further analysis to assess potential risk factors, HBsAg was negative, HCVAb was positive, HCV RNA was also positive with 500,000 IU/ML, and Alphafetoprotein (AFP) was raised and it was reported to be >1210 g/dl. There is 8.5X6.6 cm well defined mass in the right hepatic lobe (Segment 7, white arrow) with an air-fluid level and peripheral mottled gas. The underlying liver parenchyma appeared normal.

Conclusion: Gas forming ‘pyogenic’ Hepatic abscess.

Hyper-vascular liver lesion 91X94 mm (see white arrow at the same site of the different images) which shows avid enhancement and washout in subsequent phases

Conclusion: Hepatocellular Cancer

## Discussion

Liver abscess mainly results from bacterial, fungal, Tuberculosis or parasitic infections. The liver parenchyma is protected by a capsule from surrounding organs, yet infection can spread through the biliary tree, hepatic vein or through portal veins, by extension of an adjacent infection, or a result of trauma. The most common underlying condition in pyogenic liver abscess is biliary tract disease. Ascending infection can occur from billiard tract to the liver and such infections can occur sporadically or iatrogenic trauma. The incidence of liver abscess is poorly reported in Sub-Saharan Africa. Unless patients are provided with early diagnosis, followed by identification of etiologies, medical and interventional therapies as indicated, it is associated with high mortality in untreated patients (1).

Underlying hepato-biliary or pancreatic disease, liver transplantation, immunocompromising from infection with HIV/AIDS, Diabetes Mellitus is considered as the main predisposing factors (2). Our case had an underlying Diabetes Mellitus which could have predisposed him to liver abscess. Generally, liver abscess is commonly manifested on the right lobe of the liver. The right lobe of the liver has the biggest blood supply as it is supplied from the right portal vein, which is the major supply to the liver.

HCC is the fifth most common cancer and the second leading cause of cancer-related death globally (3). The presence of liver malignancies in patients with liver abscess is quite rare. The potential mechanisms could be an initial cancer which undergoes necrosis, followed by superinfection or secondary obstruction of the biliary system by the tumor or compression due to surrounding lymph nodes (4). In such cases, patients will initially present with liver cancer which later complicates with an abscess and not vice versa.

A study comprising review of 32,254 cases of liver abscess over a 12 years evaluation, has shown that, from all cases diagnosed with liver abscess, only 2% of the cases present with an initial manifestation of Hepatocellular cancer. The Liver abscess were diagnosed early in the course of the illness and Hepatitis C was an important predictor of such presentation. The study has also reviewed prognosis of such patients and it has concluded that patients who present initially with a liver abscess have the poorest outcome (5).

Another case series of 10 Taiwan patients with HCC presenting initially with liver abscess have also established that the prognosis was dismal with a mean survival of 3.5 months (8 days-6 months). The presence of pyogenic liver abscess heralds subsequent cancer risk. In this large-scale population-based study, patients with PLA were monitored and after a median followup of 3.33 +/- 3.45 years, 186 (15%) were diagnosed with different forms of cancers where, one-third of the malignancies were due to liver cancers (6).

These studies have shown an association between PLA and HCC risk and also revealed an increased risk and incidence of delayed-onset HCC in patients with liver abscess.

To the author's knowledge, there are no reports of primary liver cancer initially presenting as a liver abscess in Africa. We have also observed that patients with liver abscess are treated with antibiotics and discharged from care after percutaneous drainage.

We recommend work up of patients for liver cancer while presenting with liver abscess. The presence of risk factors, including Hepatitis C virus and Diabetes Mellitus are important predictors Oliver cancer. The presence of risk factors, together with raised AFP and abdominal CT characteristics confirms the diagnosis of HCC. I

Pyogenic liver abscess could be an initial manifestation of a deadly hepato-cellular cancer. Clinicians treating patients with liver abscess should be cognizant of the possibility of liver cancer in patients with underlying risk factors.
